# 

**Published:** 1880-11-20

**Authors:** 


### TABLE OF CONTENTS.

ORIGINAL AND SELECTED ARTICLES.

Common Earache, by Thomas F. Houston, M.D., Atlanta, Ga., 393. A New Aural Douche, by C. R. Upson, Surgeon to the Department of Nose, Throat and Lung Diseases, Atlanta Hospital, Atlanta, Ga., 396. Chronic Pharyngitis accidentally, yet successfully Treated by a New Process of Cauterization, by J. S. Stidham, M.D., of Georgia, 397. Chloroform Poisoning, treated with Hypodermic Injections of Strychnia and Whiskey, by W. Perrin Nicolson, M.D., Professor of Anatomy in Southern Medical College, Atlanta, Ga., 398. Treatment of Typhoid Fever in the Philadelphia Hospitals, 399. A Case of Puerperal Convulsions—Death, 403. Ergot-Poisoning, by John M. Keating, M.D., 406. Cholera Infantum, by Dudley M. Culver, M.D., Indiana, 407. The Elastic Ligature in Fistula in Ano, 408.

ABSTRACTS AND GLEANINGS.

Ophthalmia Neonatorum, 409. Gangrene from the Use of Ergot, 410. Prevention of the Spread of Typhoid Fever, 411. Treatment of Hemorrhoids by "Crushing," 414. Transplantation of Skin from the Sheep to the Human Body; Treatment of Diphtheria, 414. Iodine Treatment of Intermittent fever, 415. Uses of Boracic Acid, 416. Syphilis in Relation to Marriage, 417. Non-Specific Treatment of Gonorrhœa, 418. Prussian Blue in Malarial Fever, 419. The Coca in Opium Habit; Cause and Treatment of Bromidosis of the Feet, 420. Hysterics; Boracic Acid Injections in Gonorrhœa; Transverse Depressions on the Nails, 421. Hodge Pessary; Concerning Coca; Post-Nasal Catarrh, 422.

SCIENTIFIC ITEMS.

Science and Spiritualism; Self-Winding Clocks, 423. Structure of Spermatozoa; Prof. Jager's Theory of Man; Atoms, 424.

PRACTICAL NOTES AND FORMULÆ.

Compound Syrup of Hypophosphites; Nitrite of Amyl in Uterine Hemorrhage, 425. Cuticura Resolvent; How to Make a Poultice; Odorless Iodoform, 426. Soothing Ointment; Erysipelas; Abrasions of the Cornea, 427. Arsenic in Neuralgia; Chloral in the Vomiting of Pregnancy; Wound Dressing; Venereal Warts; Waterproof Varnish, 428.

EDITORIAL AND MISCELLANEOUS.

Editorial Notices, 429. National Board of Health vs. Louisiana State Board of Health; Book Notices, 430. Special Notices, 432.

